# Prediction and Classification of Alzheimer’s Disease Based on Combined Features From Apolipoprotein-E Genotype, Cerebrospinal Fluid, MR, and FDG-PET Imaging Biomarkers

**DOI:** 10.3389/fncom.2019.00072

**Published:** 2019-10-16

**Authors:** Yubraj Gupta, Ramesh Kumar Lama, Goo-Rak Kwon, Alzheimer's Disease Neuroimaging Initiative, Michael W. Weiner

**Affiliations:** Author Affiliations: UC San Francisco; University of Southern California; UC San Francisco University of Southern California Mayo Clinic, Rochester Mayo Clinic, Rochester; UC Berkeley; U Pennsylvania; USC; UC Davis; Brigham and Women's Hospital/Harvard Medical School Indiana University Washington University St. Louis University of Pennsylvania; Prevent Alzheimer's Disease 2020 (Chair) Siemens; Alzheimer's Association University of Pittsburgh Washington University St. Louis Cornell University; Albert Einstein College of Medicine of Yeshiva University; AD Drug Discovery Foundation; Acumen Pharmaceuticals; Washington University St. Louis; Northwestern University; National Institute of Mental Health; Brown University; Eli Lilly (Chair); BWH/HMS (Chair); University of Washington (Chair); Mayo Clinic, Rochester (Core PI) University of Southern California; UC San Diego; UC San Diego; UC San Diego; UC San Diego; UC San Diego; UC San Diego; UC San Diego; UC San Diego; UC San Diego; UC Davis (Core PI); UC Davis; UC San Diego; Mayo Clinic, Rochester (Core PI); Mayo Clinic, Rochester; University of London; UCLA School of Medicine; UCSF MRI; UC Davis; Mayo Clinic; Mayo Clinic; Mayo Clinic; Mayo Clinic; Mayo Clinic; Mayo Clinic; Mayo Clinic; UC Berkeley (Core PI); University of Michigan; University of Utah; Banner Alzheimer's Institute; Banner Alzheimer's Institute; University of Pittsburgh; UC Berkeley; Washington University St. Louis; Washington University St. Louis; Washington University St. Louis; Washington University St. Louis; UPenn School of Medicine; UPenn School of Medicine; UPenn School of Medicine; UPenn School of Medicine; UPenn School of Medicine; USC (Core PI); USC; USC; Indiana University; Indiana University; UC Irvine; Indiana University; Indiana University; Indiana University; Indiana University; UC San Francisco; UC San Diego; Prevent Alzheimer's Disease 2020; UC San Diego; National Institute on Aging; UC San Francisco; Brown University; National Institute of Mental Health; Cornell University; Johns Hopkins University; Richard Frank Consulting; Prevent Alzheimer's Disease 2020; National Institute on Aging; Oregon Health & Science University; University of Southern California; University of California - San Diego; University of Michigan; Mayo Clinic, Rochester; Baylor College of Medicine; Columbia University Medical Center; Washington University, St. Louis; University of Alabama - Birmingham; Mount Sinai School of Medicine; Rush University Medical Center; Wien Center; Johns Hopkins University; New York University; Duke University Medical Center; University of Pennsylvania; University of Kentucky; University of Pittsburgh; University of Rochester Medical Center; University of California, Irvine; University of Texas Southwestern Medical School; Emory University; University of Kansas, Medical Center; University of California, Los Angeles; Mayo Clinic, Jacksonville; Indiana University; Yale University School of Medicine; McGill Univ., Montreal-Jewish General Hospital; Sunnybrook Health Sciences, Ontario; U.B.C. Clinic for AD & Related Disorders; Cognitive Neurology - St. Joseph's, Ontario; Cleveland Clinic Lou Ruvo Center for Brain Health; Northwestern University; Premiere Research Inst (Palm Beach Neurology); Georgetown University Medical Center; Brigham and Women's Hospital; Stanford University; Banner Sun Health Research Institute; Boston University; Howard University; Case Western Reserve University; University of California, Davis - Sacramento; Neurological Care of CNY; Parkwood Hospital; University of Wisconsin; University of California, Irvine - BIC; Banner Alzheimer's Institute; Dent Neurologic Institute; Ohio State University; Albany Medical College; Hartford Hospital, Olin Neuropsychiatry Research Center; Dartmouth-Hitchcock Medical Center; Wake Forest University Health Sciences; Rhode Island Hospital; Butler Hospital; UC San Francisco; Medical University South Carolina; St. Joseph's Health Care; Nathan Kline Institute; University of Iowa College of Medicine; Cornell University; University of South Florida: USF Health Byrd Alzheimer's Institute; University of California, San Francisco; University of Southern California; UC San Francisco; University of Southern California; Mayo Clinic, Rochester; Brigham and Women's Hospital/ Harvard Medical School; UC Davis; Mayo Clinic, Rochester; UC Berkeley; Washington University St. Louis; Indiana University; Perelman School of Medicine, UPenn; USC; Perelman School of Medicine, University of Pennsylvania; UC San Francisco; Rehabilitation Institute of Chicago, Feinberg School of Medicine, Northwestern University; BWH/HMS (Chair); University of Washington (Chair); Core PI; Mayo Clinic, Rochester (Core PI); University of Southern California; UC San Diego; UC San Diego; UC San Diego; UC San Diego; UC San Diego; UC San Diego; UC San Diego; UC San Francisco; UC San Francisco; UC San Francisco; UC Davis (Core PI); UC San Diego; Mayo Clinic, Rochester (Core PI); Mayo Clinic, Rochester; Mayo Clinic; Mayo Clinic; Mayo Clinic; Mayo Clinic; Mayo Clinic; UC Berkeley (Core PI); University of Michigan; University of Utah; Banner Alzheimer's Institute; Banner Alzheimer's Institute; UC Berkeley; Washington University St. Louis; Washington University St. Louis; Washington University St. Louis; Perelman School of Medicine, UPenn; Perelman School of Medicine, UPenn; Perelman School of Medicine, UPenn; Perelman School of Medicine, UPenn; Perelman School of Medicine, UPenn; USC (Core PI); USC; USC; Indiana University; Indiana University; UC Irvine; Indiana University; Indiana University; Indiana University; Indiana University; UC San Francisco; Department of Defense (retired); University of Southern California; University of California, San Diego; Columbia University Medical Center; Rush University Medical Center; Wien Center; Duke University Medical Center; University of Rochester Medical Center; University of California, Irvine; Medical University South Carolina; Premiere Research Inst (Palm Beach Neurology); University of California, San Francisco; Georgetown University Medical Center; Brigham and Women's Hospital; Banner Sun Health Research Institute; Howard University; University of Wisconsin; University of Washington; Stanford University; Cornell University; ADNI Depression; Principal Investigator; University of California, San Francisco; ATRI PI and Director of Coordinating Center Clinical Core; University of Southern California; University of Southern California; Executive Committee; UC San Francisco; UC San Francisco; University of Southern California; University of Southern California; Mayo Clinic, Rochester; UC Berkeley; Indiana University; University of Southern California; UC Davis; University of Michigan; Data and Publication Committee (DPC); BWH/HMS (Chair); BWM/HMS (Director); Clinical Core Leaders; Core PI; University of Southern California; University of Southern California; University of Southern California; Clinical Informatics, Operations and Regulatory Affairs; USC; USC; USC; USC; USC; USC; USC; Psychiatry Site Leaders and Key Personnel; UC San Francisco; UC San Francisco; UC San Francisco; University of Pittsburgh; University of Pittsburgh; MRI Core Leaders and Key Personnel; Mayo Clinic, Rochester (Core PI); Mayo Clinic, Rochester; Mayo Clinic, Rochester; Mayo Clinic, Rochester; Mayo Clinic, Rochester; Mayo Clinic, Rochester; Mayo Clinic, Rochester; Mayo Clinic, Rochester; PET Core Leaders and Key Personnel; University of Michigan; UC Berkeley; Informatics Core Leaders and Key Personnel; USC (Core PI); USC; USC; Genetics Core Leaders and Key Personnel; Indiana University; Indiana University; Indiana University; Indiana University; Indiana University; University of California, San Francisco: University of Pittsburgh; Department of Information and Communication Engineering, Chosun University, Gwangju, South Korea

**Keywords:** Alzheimer's disease, MCIs (MCI stable), MCIc (MCI converted), sMRI, FDG-PET, CSF, apolipoprotein-E (APOE) genotype, support vector machine

## Abstract

Alzheimer's disease (AD), including its mild cognitive impairment (MCI) phase that may or may not progress into the AD, is the most ordinary form of dementia. It is extremely important to correctly identify patients during the MCI stage because this is the phase where AD may or may not develop. Thus, it is crucial to predict outcomes during this phase. Thus far, many researchers have worked on only using a single modality of a biomarker for the diagnosis of AD or MCI. Although recent studies show that a combination of one or more different biomarkers may provide complementary information for the diagnosis, it also increases the classification accuracy distinguishing between different groups. In this paper, we propose a novel machine learning-based framework to discriminate subjects with AD or MCI utilizing a combination of four different biomarkers: fluorodeoxyglucose positron emission tomography (FDG-PET), structural magnetic resonance imaging (sMRI), cerebrospinal fluid (CSF) protein levels, and Apolipoprotein-E (APOE) genotype. The Alzheimer's Disease Neuroimaging Initiative (ADNI) baseline dataset was used in this study. In total, there were 158 subjects for whom all four modalities of biomarker were available. Of the 158 subjects, 38 subjects were in the AD group, 82 subjects were in MCI groups (including 46 in MCIc [MCI converted; conversion to AD within 24 months of time period], and 36 in MCIs [MCI stable; no conversion to AD within 24 months of time period]), and the remaining 38 subjects were in the healthy control (HC) group. For each image, we extracted 246 regions of interest (as features) using the Brainnetome template image and NiftyReg toolbox, and later we combined these features with three CSF and two APOE genotype features obtained from the ADNI website for each subject using early fusion technique. Here, a different kernel-based multiclass support vector machine (SVM) classifier with a grid-search method was applied. Before passing the obtained features to the classifier, we have used truncated singular value decomposition (Truncated SVD) dimensionality reduction technique to reduce high dimensional features into a lower-dimensional feature. As a result, our combined method achieved an area under the receiver operating characteristic (AU-ROC) curve of 98.33, 93.59, 96.83, 94.64, 96.43, and 95.24% for AD vs. HC, MCIs vs. MCIc, AD vs. MCIs, AD vs. MCIc, HC vs. MCIc, and HC vs. MCIs subjects which are high relative to single modality results and other state-of-the-art approaches. Moreover, combined multimodal methods have improved the classification performance over the unimodal classification.

## Introduction

Alzheimer's disease (AD) is an age-related neurodegenerative disorder that is commonly seen in the aging population. Its prevalence is expected to increase greatly in the coming years as it affects one out of nine people over the age of 65 years (Bain et al., 2008). AD involves progressive cognitive impairment, commonly associated with early memory loss, leading patients to require assistance for activities of self-care during its advanced stages. AD is characterized by the accumulation of amyloid-beta (Aβ) peptide in amyloid plaques in the extracellular brain parenchyma and by intra-neuronal neurofibrillary tangles caused by the abnormal phosphorylation of the tau protein (De Leon et al., 2007). Amyloid deposits and tangles are necessary for the postmortem diagnosis of AD. A prediction of an AD dementia in a predictable time-period, i.e., within 1–2 years, appears much more pertinent in a clinical outlook than a prediction of AD dementia in the faraway future, e.g., in 10–20 years. Individual classified to be at “short-term risk” can receive more active treatment and counseling. Mild cognitive impairment (MCI) is a prodromal (predementia) stage of AD, and recent studies have shown that individuals with amnestic MCI tend to progress to probable AD at a rate of ~10–15% per year (Braak and Braak, 1995; Braak et al., 1998). Thus, accurate diagnosis of AD, and especially MCI, is of great size for prompt treatment and likely delay of the progression of the disease. MCI patients who do not progress to AD either develop another form of dementia, retain a stable condition or revert to a non-demented state. Therefore, predicting which MCI patients will develop AD in the short-term and who will remain stable is extremely relevant to future treatments and is complicated by the fact that both AD and MCI affect the same structures of the brain. In subjects with MCI, the effects of cerebral amyloidosis and hippocampal atrophy on the progression to AD dementia differ, e.g., the risk profile is linear with hippocampal atrophy but reaches a ceiling with higher values for cerebral amyloidosis (Jack et al., 2010). In subsequent investigations, biomarkers of neural injury appeared to best predict AD dementia from MCI subjects at shorter time intervals (1–2 years) in particular (Dickerson, 2013). This demonstrates the great importance of developing a sensitive biomarker that can detect and monitor early changes in the brain. The ability to diagnose and classify AD or MCI at an early stage allows clinicians to make more knowledgeable decisions at a later period regarding clinical interventions or treatment planning, thus having a great impact on reducing the cost of longtime care.

Over the past several years, several classification methods have been implemented to overcome these problems using only a single modality of biomarkers. For example, many high-dimensional classification techniques use only the sMR images for classification of AD and MCI. sMRI captures the disease-related structure patterns by measuring the loss of brain volumes and decreases in cortical thickness (Davatzikos et al., 2008; Cuingnet et al., 2011; Salvatore et al., 2015; Beheshti et al., 2016, 2017; Jha et al., 2017; Lama et al., 2017; Long et al., 2017) for the early prediction of AD and MCI. A number of studies, covering volume of interest, region of interest (ROI), shape analysis and voxel-based morphometry, have reported that the amount of atrophy in several sMRI brain regions, such as the entorhinal cortex, hippocampus, parahippocampal gyrus, cingulate, and medial temporal cortex (Cuingnet et al., 2011; Moradi et al., 2015; Beheshti et al., 2016; Gupta et al., 2019), are sensitive to the disease progression and prediction of MCI conversion. In addition to the sMRI, another important modality of biomarkers thoroughly established neuroimaging tool in the diagnosis of neurodegenerative dementia (AD or MCI) is 18F-FDG-PET image, which mainly measures hypometabolism, reflecting neuronal dysfunction (Minoshima et al., 1997; Foster et al., 2007; Li et al., 2008; Förster et al., 2012; Nozadi et al., 2018; Samper-González et al., 2018). With FDG-PET image, some recent studies have reported the reduction of glucose metabolism or an alternations of hypometabolism occurs in the posterior cingulated cortex, precuneus, and posterior parietal temporal association cortex (Förster et al., 2012), and it usually precedes cortical atrophy (Minoshima et al., 1997; Li et al., 2008) and clinical cognitive symptoms in AD patients. Besides these neuroimaging biomarkers, there are also some biochemical (blood-protein level) and genetic (gene-protein level) biomarkers for the diagnosis of AD and MCI subjects. Biochemical changes in the brain are reflected in the cerebrospinal fluid (CSF) (Chiam et al., 2014; Zetterberg and Burnham, 2019), decreased CSF levels of amyloid-beta (Aβ) 1 to 42 peptide (Aβ_1−−42_; a marker of amyloid mis-metabolism) (Blennow, 2004; Shaw et al., 2009; Frölich et al., 2017), and elevations of total tau (t-tau) and hyperphosphorylated tau at the threonine181 (p-tau_181p_) protein (markers of axonal damage and neurofibrillary tangles) (Andreasen et al., 1998; Anoop et al., 2010; Fjell et al., 2010), are considered to be CSF best established predictive biomarkers of AD dementia in patients with MCI. Recent studies have shown that alternation or reduction of polymorphism (genetics) also play a vital role in AD and MCI patients (Gatz et al., 2006; Spampinato et al., 2011; Dixon et al., 2014). Perhaps, the most commonly considered polymorphism in cognitive and neurodegenerative aging is apolipoprotein E (APOE; rs7412; rs429358). It involved in lipid transfer, cell metabolism, repair of neuronal injury due to oxidative stress, amyloid-beta peptide accumulation, and in elderly process. A gene on chromosome 19 in a locus synthesizes APOE with three alleles (ε2, ε3, and ε4) and it is expressed in the central nervous system in astrocytes and neurons. The APOE ε4 allele has been consistently linked to normal cognitive decline in MCI and AD dementia patients (Luciano et al., 2009; Brainerd et al., 2011; Alzheimer's Disease Neuroimaging Initiative et al., 2016; Sapkota et al., 2017). It is also said that especially APOE ε4 is the strongest genetic risk factor that increases the occurrence with a 2-to 3-fold risk for AD, and it lowers the age of onset AD. These all research focuses using only a single modality of biomarkers and their proposed algorithm performance is low compared to a recently published multimodal method (Zhang et al., 2011; Suk et al., 2014; Ritter et al., 2015; Frölich et al., 2017; Li et al., 2017; Gupta et al., 2019). These studies suggest that classification performance will improve when combining all different modalities of biomarkers into one form because different biomarkers offer a piece of different complementary information (or capture disease information from different outlooks) which are useful for the early classification of the AD and MCI patients.

Recently, Jack et al. (2016, 2018) proposed the A/T/N system, as shown in Table 1, in which seven major AD biomarkers are divided into three binary categories based on the nature of the pathophysiology that each subject exhibits.

**Table 1:** A/T/N biomarker grouping.

**A**	**T**	**N**
Aggregated Aβ or associated pathological state	Aggregated tau (neurofibrillary tangles) or associated pathological state	Neurodegeneration or neural injury
CSF Aβ42, or Aβ42/Aβ40 ratio	CSF phosphorylated tau	Anatomical MRI
Amyloid PET	Tau PET	FDG-PET, CSF total tau

Based on the above system, we propose to combine four different modalities of biomarkers, fluorodeoxyglucose positron emission tomography (FDG-PET), structural magnetic resonance imaging (sMRI), cerebrospinal fluid (CSF) protein levels, and the apolipoprotein E (APOE) genotype, of each patient. Over the past few years, several techniques have been proposed using either a combination of two or three different biomarker modalities, such as the combination of MRI and CSF biomarkers (Vemuri et al., 2009; Fjell et al., 2010; Davatzikos et al., 2011); MRI and FDG-PET biomarkers (Chetelat et al., 2007; Li et al., 2008, 2017; Shaffer et al., 2013); MRI, FDG-PET, and CSF (Walhovd et al., 2010; Zhang et al., 2011; Shaffer et al., 2013; Ahmed et al., 2014; Ritter et al., 2015); and MRI, FDG-PET, and APOE (Young et al., 2013). Although these published approaches have utilized a combination of different types of biomarkers to develop neuroimaging biomarkers for AD, the above methods may be limited. They have used brain atrophy from a few manually extracted regions as a feature for sMRI and PET images to classify different groups. However, using only a small number of brain regions as features from any imaging modality may not be able to reflect the spatiotemporal pattern of structural and physiological abnormalities in their entirety (Fan et al., 2008). Furthermore, by only increasing the number of biomarkers, their combination did not lead to an increase in predictive power. As Heister et al. (2011) explained, a combination of impaired learning ability with medial temporal atrophy was associated with the greatest risk of developing AD in a group of MCI patients.

In this study, we propose a novel approach for the early detection of AD with other groups and to differentiate the most similar clinical entities of MCIs and MCIc by combining biomarkers from two imaging modalities (sMRI, FDG-PET) with CSF (biochemical protein level) and APOE genotype biomarkers obtained from each patient. As the A/T/N system defines that each modality of biomarkers offers a different complementary information, which is useful for the early classification of AD and MCI subjects, so in our study we have used four different modalities of biomarkers, sMRI, FDG-PET, CSF (biochemical protein level), and APOE genotype for the early prediction of AD and MCI subjects. Moreover, using early fusion method we have combined the measurement from all four (sMRI, FDG-PET, CSF, and APOE) different biomarkers to discriminate between AD and HC, MCIc and MCIs, AD and MCIs, AD and MCIc, HC and MCIs, and HC and MCIc. We compare classification performance for different groups using typical measures of gray matter atrophy (from sMR image), average intensity of each region (from FDG-PET image), t-tau, p-tau_181p_, and Aβ_42_ scores (from biochemical level), and ε3/ε4, ε4/ε4 values from APOE genotype biomarker. To distinguish between these groups, we used a different kernel-based multiclass SVM classifier with a 10-fold stratified cross-validation technique that helps to find the optimal hyperparameter for this classifier. Our experiment results show that the grouping of different measurements from four different modalities of biomarkers exhibits much better performance for all classification groups than using the best individual modality of the biomarkers.

## Materials and Methods

### Participants

Data used in the preparation of this article were obtained from the Alzheimer's Disease Neuroimaging Initiative (ADNI) database (http://adni.loni.usc.edu/ADNI). The ADNI was launched in 2003 as a public-private partnership led by Principal Investigator, Michael W. Weiner, MD. The primary goal of the ADNI has been to test whether serial magnetic resonance imaging (MRI), positron emission tomography (PET), other biological markers, and clinical and neuropsychological assessment can be combined to measure the progression of mild cognitive impairment (MCI) and early Alzheimer's disease (AD). For up-to-date information, see https://www.adni-info.org.

In total, we included 158 different subjects from the ADNI database. Included subjects were African-American, Asian, and white who stay in America and their age were between 50 and 89 years and spoke either Spanish or English. Patients with specific psychoactive medications have been excluded from the study while taking scans and the general inclusion/exclusion norms were as follows: for an HC subject, a Clinical Dementia Rating (CDR) (Morris, 1993) of 0, Mini-Mental State Examination (MMSE) score must be between 24 and 30 (inclusive), non-MCI, non-depressed, and non-demented. MCI subjects had a CDR level of 0.5, MMSE scores between 24 and 30 (inclusive), a slight memory complaint, having objective memory loss measured by education adjusted scores on Wechsler Memory Scale Logical Memory II (Elwood, 1991), absence of significant levels of impairment in other cognitive domains, essentially preserved activities of daily living, and an absence of dementia, and for an AD patients the MMSE scores between 20 and 26, CDR level of 0.5 or 1.0, and meets the National Institute of Neurological and Communicative Disorders and Stroke and the Alzheimer's Disease and Related Disorders Association (NINCDS/ADRDA) criteria for probable AD. We selected all subjects for whom all four modalities of biomarkers were available. The four obtained biomarkers were 1.5-T T1-weighted sMRI, FDG-PET, CSF measures of three protein levels (t-tau, p-tau_181p_, and Aβ_42_), and APOE genotype. Of the 158 subjects, 38 subjects were in the AD group (MMSE ≤ 24), 82 subjects in the MCI group (46 with MCIc [converted to AD within 24 months of the time-period] and 36 with MCIs [patients who did not convert to AD within 24 months of the time-period]) (MMSE ≤ 28). The remaining 38 subjects were healthy controls (MMSE ≤ 30).

Table 2 shows the neuropsychological and demographic information for the 158 subjects. To measure the statistically important difference in demographics and clinical features, Student's *t*-test was applied using age data, were the significance value was set to 0.05. No any significant differences were found for any groups. In all groups, the number of male subjects was higher than the number of female subjects. Compared to the other groups, the HC group had higher scores on the MMSE. Healthy subjects had a significantly lower Geriatric Depression Scale (GDS) scores than the other groups. The Functional Activities Questionnaire (FAQ) was higher for the AD group than the other groups.

**Table 2:** Demographical and neuropsychological characteristics of the studied sample.

**Groups**	**AD**	**MCIs**	**MCIc**	**HC**
No. of Subjects	38	36	46	38
Male/female	22/16	26/10	29/17	25/13
Age	77.15^*^± 6.88	74.22^*^± 5.65	76.71^*^± 7.71	76.68^*^± 5.01
MMSE	21.21^*^± 4.45	26.91^*^± 2.43	26.19^*^± 2.79	29.05^*^± 1.23
FAQ	17.42^*^± 6.92	3.80^*^± 4.06	7^*^± 5.90	0.315^*^± 1.02
Subject weight	73.90^*^± 13.18	78.44^*^± 14.64	73.51^*^± 13.17	74.43^*^± 14.38
GDS	1.68^*^± 1.52	1.58^*^± 1.58	1.63^*^± 1.50	0.86^*^± 1.12

* *Values are presented as mean ± and standard deviation (SD)*.

### MRI and FDG-PET Datasets

#### MRI Protocol

Structural MRI scans were acquired from all data centers using Philips, GE, and Siemens scanners. Since the acquisition protocols were different for each scanner, an image normalization step was performed by the ADNI. The imagining sequence was a 3-dimensional sagittal part magnetization prepared of rapid gradient-echo (MPRAGE). This sequence was repeated consecutively to increase the likelihood of obtaining at least one decent quality of MPRAGE image. Image corrections involved calibration, geometry distortion, and reduction of the intensity of non-uniformity applied on each image by the ADNI. More details concerning the sMRI images is available on the ADNI homepage (http://adni.loni.usc.edu/methods/mri-tool/mri-analysis/). We used 1.5-T sMRI T1-weighted images from the ADNI website. Briefly, raw (NIFTY) sMRI scans were downloaded from the ADNI website. All scans were 176 × 256 × 256 resolution with 1 mm spacing between each scan.

#### FDG-PET Protocol

The FDG-PET dataset was acquired from the ADNI website. A detailed explanation of the FDG-PET image acquisition is available on the ADNI homepage (http://adni.loni.usc.edu/pet-analysis-method/pet-analysis/). Briefly, FDG-PET images were acquired from 30 to 60 min post-injection. First, images were averaged and then spatially aligned. Next, these images were interpolated to a standard voxel size, and later intensity normalization was performed. Finally, images were smoothed to a common surface of 8 mm (FWHM) full width at half maximum. First, the FDG-PET images were downloaded in the Digital Imagining and Communication in Medicine (DICOM) format. In the second step, we use the dcm2nii (Li et al., 2016) converter to convert DICOM images into the Nifty format. All scans were 160 × 160 × 96 resolution with 1.5 mm spacing between each scan.

### CSF and APOE Genotype

#### CSF

We downloaded the required CSF biomarker values for each selected MRI and FDG-PET image from the ADNI website. A brief description regarding the collection procedure is available on the ADNI website. As the manual describes, a 20-ml volume was obtained from each subject using a lumbar puncture with a 24 or 25 gauge atraumatic needle around the time of their baseline scans. Subsequently, all samples were stored on dry ice on the same day and later they were sent to the University of Pennsylvania AD Biomarker Fluid Bank Laboratory where the levels of proteins (Aβ_42_, total tau, and phosphorylated tau) were measured and recorded. In this study, the three protein levels, Aβ_42_, t-tau, and p-tau_181p_, were used as features.

#### APOE Genotype

APOE genotype is known to affect the risk of developing sporadic AD in carriers. Basically, there are three types of the APOE gene, called alleles: APOE_2_, APOE_3_, and APOE_4_. Everyone has two copies of gene and their combination (ε2/ε2, ε2/ε3, ε2/ε4, ε3/ε3, ε3/ε4, and ε4/ε4) determines our APOE genotype score. The APOE (ε2) allele is the rarest form of APOE and carrying even one copy appears to reduce the risk of developing AD by up to 40%. APOE (ε3) is the most common allele and doesn't seem to influence risk whereas APOE (ε4) allele which present in ~10–15% of people, and having one copy of ε4 (ε3/ε4) can increase the risk of having AD by 2–3 times while having the two copies (ε4/ε4) of APOE ε4 can increase the risk by 12 times. The APOE genotype of each subject was recorded as a pair of numbers representing which two alleles were present in the blood. The APOE genotype was obtained from 10 ml of a blood sample taken at the time of the scan and sent immediately to the University of Pennsylvania AD Biomarker Fluid Bank Laboratory for analysis. The APOE genotype value was available for all subjects for whom we had imagining data.

### Overview of Proposed Framework

**Figure 1:**
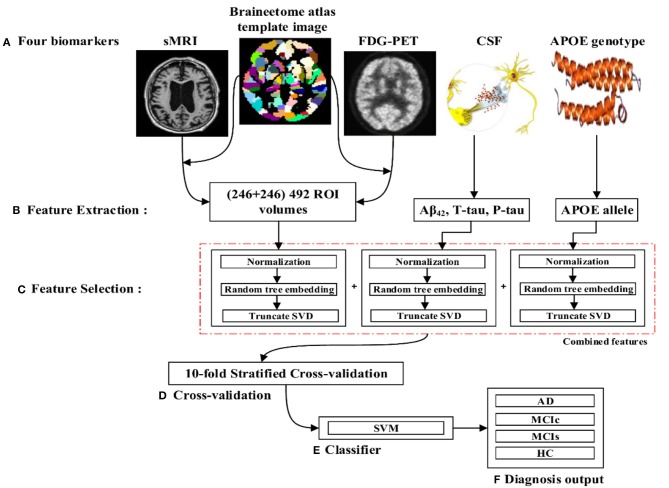
Overview of the proposed framework. **(A)** Selection of four (sMRI, FDG-PET, CSF, and APOE) important biomarkers. **(B)** Feature extraction using NiftyReg toolbox for sMRI and FDG-PET image. **(C)** Feature selection using truncate SVD method. **(D)** Ten-fold stratified cross-validation method. **(E)** Kernel-based multiclass SVM classifier. **(F)** Diagnosis output.

The proposed framework consists of three processing stages: feature extraction and fusion of multiple features into the single form using early fusion technique, optimal feature subset selection using truncate SVD dimensionality reduction method, and classification. Figure 1 illustrates the block diagram of the proposed framework. The set of participants were randomly split into two groups in a 75:25 ratios as a training and testing sets, respectively, before passing them to the kernel-based multiclass SVM classifier. Moreover, during the training stage, a gray matter atrophy (from sMR image) and average intensity of each region (from FDG-PET image) which had automatically extracted using NiftyReg toolbox, as well as a set of t-tau, p-tau_181p_, and Aβ_42_ (from biochemical level) CSF scores, and (ε3/ε4, ε4/ε4) values from APOE genotype biomarker, were downloaded from the ADNI website. Here, we have used random tree embedding (Geurts et al., 2006; Moosmann et al., 2008) method to transform low dimensional data into a higher dimensional state, to make sure that the complementary information found across all modalities is still used while classifying AD subjects. In addition, we have used an early fusion technique for the combination of different features into one form before passing them to the feature selection process. Moreover, a feature selection technique using truncate SVD was employed to select the optimal subsets of features from the bunch of features, including the sMRI, FDG-PET, CSF, and APOE extracted features to train the classifiers to distinguish between AD and HC, MCIc and MCIs, AD and MCIs, AD and MCIc, HC and MCIs, and HC and MCIc groups. In the testing stage, a remaining 25% of the dataset is then passed to the kernel-based multiclass SVM classifier to measure the performance of our proposed method.

### Image Analysis and Feature Extraction

**Figure 2:**
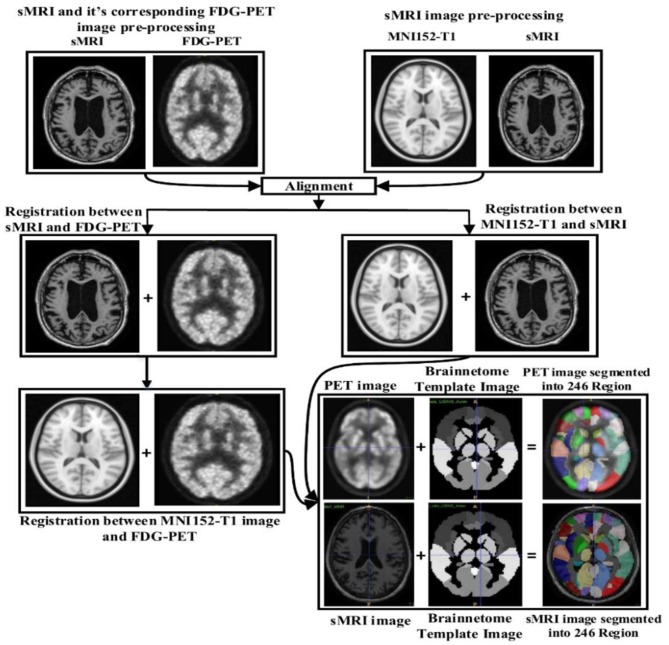
Overview of the feature extraction pipeline for sMRI and FDG-PET image. Here, NiftyReg toolbox is used for the image registration and as well as for the non-linear registration between the (sMRI and FDG-PET) image with 2 mm Brainnetome template image. Above pipeline shows that, we have successfully extracted 246 ROI's from each (sMRI and FDG-PET) images.

Image preprocessing was performed for all sMR and FDG-PET images. First, we performed anterior commissure (AC)–posterior commissure (PC) correction for all subjects. Afterward, we used N4 bias field correction using ANTs toolbox (Tustison et al., 2010) to correct the intensity of inhomogeneity for each image. In our pipeline, skull striping was not necessary as images were already preprocessed. Therefore, we reduced the total number of required pre-processing steps for the original images. Later high-dimensional data from the images were preserved for the feature extraction step. For sMR images, we first aligned them to the MNI152 T1-weighted standard image using SPM12 (Ashburner and Friston, 2000) toolbox in Matlab 2018b. For the purpose of anatomical segmentation or parcellation of whole-brain into anatomic regions and to quantify the features of each specific regions of interest (ROI) from each sMR image, we have used NiftyReg toolbox (Modat et al., 2010) with 2-mm Brainnetome atlas template (Fan et al., 2016) image, which is already segmented into 246 regions, 210 cortical and 36 subcortical regions. Moreover, we processed the sMRI image using open source software, NiftyReg (Modat et al., 2010), which is an automated registration toolkit that performs fast diffeomorphic non-rigid registration. After the registration process, we gained the subject-labeled image based on a 2-mm Brainnetome atlas template with 246 segmented regions. For the 246 ROI in the labeled sMR images, we computed the volume of gray matter tissues in that ROI and used it as a feature. For the FDG-PET images, the first step was to register the FDG-PET image to its corresponding sMRI T1-weighted image, using the reg_aladin command from the NiftyReg software. Once the FDG-PET images were registered with their respective MR images, we again used NiftyReg toolbox for non-rigid registration between processed FDG-PET image and the 2-mm Brainnetome atlas template image. After registration, we obtained 246 segmented regions for each FDG-PET image. Again, we computed the average intensity of each region for the ROI and used it as a feature for classification. Figure 2 shows the pipeline for extraction of 246 regions from sMRI and FDG-PET image.

Therefore, for each subject, we obtained 246 ROI's features for each sMRI image, another 246 features for each FDG-PET image. Three features from CSF biomarkers for each subject, and two feature values from APOE genotype for all selected images.

### Combining Multimodality of Biomarkers

**Figure 3:**
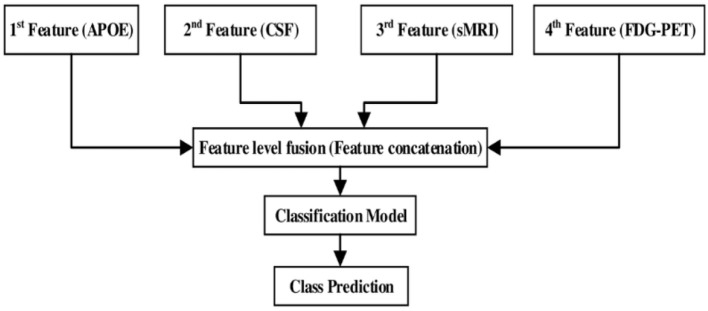
Multimodal fusion pipeline shows the fusion of four modality of biomarkers, from which two biomarkers sMRI and FDG-PET belongs to imaging modality, CSF biomarker from biochemical, and APOE biomarker from genetics.

After assessing the performance for each individual modality, we combined different modalities in order to study possible improvements in classification performance. Here, a general framework based on an early fusion (or straightforward feature concatenation) method which use special combination rules to combine (or to concatenate) complementary information from different modalities of biomarker into single feature vector is used, and later we have used kernel-based multiclass SVM classifier to train that single feature vector. In this context, various authors have combined sMRI-based features with the features calculated from FDG-PET, DTI, and fMRI (Zhang et al., 2011, 2012; Young et al., 2013; Schouten et al., 2016; Bron et al., 2017; Bouts et al., 2018) for early classification of AD subjects. Moreover, in our case, we have combined four (sMRI, FDG-PET, CSF, and APOE) modality of biomarkers into one form using early fusion technique for the early classification of AD and MCI subjects. Here, the value of the features for the APOE and CSF are of small dimensional compared to the sMRI and FDG-PET extracted features values. Therefore, if classification algorithms trained on (high + low) dimensional combined features then it may produce prediction models that effectively ignore the low dimensional features. Moreover, to overcome this problem, we have transformed low dimensional extracted features into a high dimensional state using random tree embedding method, which ensures that the complementary information found across all modalities is still used while classifying several groups. This step is followed for every classification problem. Figure 3 shows the early fusion pipeline. Moreover, here 1st (APOE), 2nd (CSF), 3rd (sMRI), and 4th (FDG-PET) features are concatenated with each other using early fusion technique before passing them further. We assessed the classification performance for individual and combined modalities by calculating the AUC for each group.

### Feature Selection

With the help of automated feature extraction methods, we extracted 246 ROIs from each sMRI and FDG-PET image. As in the neuroimaging analysis, the number of features per subjects is very high relative to the number of patients, a phenomenon normally referred to as the curse of dimensionality. Furthermore, because of the computational difficulties of dealing with high dimensional data, dealing with many features can be a challenging task, which may result in overfitting. Feature selection is an additional helpful stage prior to the classification problem, which helps to reduce the dimensionality of a feature by selecting proper features and omitting improper features. This step helps to speed up the classification process by decreasing computational time for the training and testing datasets and increases the performance of classification accuracy. At first, we normalized the extracted features using the standard scalar function from Scikit-learn library (0.19.2) (Pedregosa et al., 2011), which transforms the dataset in such way that its distribution will have a mean of 0 and unit variance of 1 to reduce the redundancy and dependency of the data. After that, we performed high dimensional transformation of the data using random tree embedding (Geurts et al., 2006; Moosmann et al., 2008) from Scikit-learn library (0.19.2) (Pedregosa et al., 2011) and a dimensionality reduction process using truncated singular value decomposition (SVD) method. Random tree embedding system works based on the decision tree ensemble learning (Brown, 2016) system that execute an unsupervised data transformation algorithm to solve a random tree embedding task. It uses a forest of complete random trees, that encodes the data by the indices of the leaves where a data sample point ends up. This index is then encoded in a one-of-k encoder, which maps the data into a very high-dimensional state which might be beneficial for the classification process. The mapping process is completely unsupervised and very efficient for any dataset. After mapping the dataset into the very high dimensional state, we applied the truncated SVD function for dimensionality reduction purposes, which only selects the important features from the complete set of features. The truncated SVD is similar to principal component analysis (PCA) but differs in that it works on the sample matrices *X* directly instead of working on their covariance matrices. When performed column-wise (per-feature), i.e., means of *X* are deducted from the value of the feature, the truncated SVD of the resulting matrix corresponds to PCA. Truncated SVD implements an irregular SVD that only calculates the *k* largest singular values, where *k* is a user-specified parameter. Mathematically, the truncated SVD can be applied to train data *X*, which produces a low-rank approximation of *X*:

(1) $$\begin{array}{l} X=X_{k}=U_{k}\Sigma_{k}V_{k}^{T} \end{array}$$

After this process, $U_{k}\Sigma_{k}^{T}$ is transformed into the training set with *k* features. To transform a test set *X*, we can multiply it by *V*_*k*_:

(2) $$\begin{array}{l} X^{\prime }=XV_{k} \end{array}$$

In this way, we can perform the truncated SVD method on the training and testing dataset.

### Classification

#### Support Vector Machine

SVM is a supervised learning method. SVM (Cortes and Vapnik, 1995) works by finding a hyperplane that best separates two data groups. It is trained by training data in n-dimensional training space after which test subjects are classified according to their position in n-dimensional feature space. It has been used in several neuroimaging areas (Cui et al., 2011; Zhang et al., 2011; Young et al., 2013; Collij et al., 2016) and is known to be one of the most powerful machine learning tools in the neuroscience field in recent research. In mathematical representation, for a 2D space, a line can discriminate the linearly separable data. The equation of a line is *y* = *ax* + *b*. By renaming *x* with *x*_1_ and *y* with *x*_2_, the equation will change to *a*(*x*_1_ − *x*_2_) + *b* = 0. If we stipulate *X* = (*x*_1_, *x*_2_) and *w* = (*a*, − 1), we get *w*.*x* + *b* = 0, which is an equation of hyperplane. The linearly separable output with the hyperplane equation has the following form:

(3) $$\begin{array}{l} f\left(y\right)=z^{T}\emptyset .\left(y\right)+b \end{array}$$

Where y is an input vector, *z*^*T*^is a hyperplane parameter, and ∅(*y*) is a function used to map feature vector *y* into a higher-dimensional space. The parameters *z* and *b* are scaled suitably by the same quantity, the decision hyperplane given by the Equation (2) remains unchanged. Moreover, in order to make any decision boundary surface (hyperplane) correspond to the exclusive pair of (*z, b*), the following constraints are familiarized:

(4) $$\begin{array}{l} min|z^{T}\emptyset .\left(y_{i}\right)+b|=1,\;i=1,\ldots .,N, \end{array}$$

Where *y*_1_, *y*_2_, *y*_3_, …., *y*_*N*_ are the given training points. Equation (4) hyperplanes are known as the canonical hyperplanes. For a given hyperplane (or decision surface) which is described with the equation;

(5) $$\begin{array}{l} z^{T}\emptyset .(y)+b=0,\text{which is same as }z^{T}\emptyset .\left(y\right)\\ \;=0(\text{ which has more dimensions}) \end{array}$$

And, for a vector *x* that does not belong to the hyperplane, the following equation is satisfied (Cortes and Vapnik, 1995, Madevska-Bogdanova et al., 2004, Cui et al., 2011):

(6) $$\begin{array}{l} z^{T}\emptyset .\left(x\right)+b=\pm s||z|| \end{array}$$

Where *s* is the distance of a point *x* to the given hyperplane. The different signs determine the vector's *x* side of the hyperplane. Therefore, the output *f*(*y*) (or predictive value) of the SVM is truly proportional to the norm of vector *z* and the distance *s(x)* from the chosen hyperplane. Moreover, in our study, we have used kernel-support vector machine, which is used to solve the non-linear problem with the use of linear classifier and involved in exchanging linearly non-separable data into linearly separable data. The idea behind this concept is linearly non-separated data in n-dimensional space might be linearly separated in higher m-dimensional space. Mathematically, the kernel is indicated as,

(7) $$\begin{array}{l} K\left(a,b\right)=\;<F\left(a\right),F\left(b\right)> \end{array}$$

Where, *K* is a kernel function and *a, b* are inputs in n-dimensional space. *F* is a mapping function which maps from n-dimensional to m-dimensional space (i.e., m > n). Moreover, in our case, we have used three different kinds of kernel function which is defined as follow:

- Polynomial type: It represents the resemblance of vectors (training samples) in a feature space over the polynomials of the original variables, allowing the learning of non-linear models. A Polynomial kernel is defined as;
  (8) $$\begin{array}{l} K\left(x,y\right)={\left(x,\;y\right)}^{d} \end{array}$$

Where *x* and *y* are vectors in the input space. *d* is the kernel parameter.

- Gaussian radial basis type: Radial basis functions mostly with Gaussian form and it is represented by;
  (9) $$\begin{array}{l} K\left(x,y\right)=\exp\left(-\frac{||x-y|{|}^{2}}{2\sigma^{2}}\right) \end{array}$$

Where, *x* and *y* are the two input samples, which represented as a feature vector in input space. ||*x* − *y*||^2^ may be seen as a squared Euclidean distance between two feature vectors. σ is a kernel parameter.

- Sigmoid type: It comes from the neural networks field, where the bipolar sigmoid function is often used as an activation function for an artificial neuron. And, it is represented by;
  (10) $$\begin{array}{l} K\left(x,y\right)=\tanh\left(\propto x^{T}y+c\right) \end{array}$$

Where, *x* and *y* are vectors in the input space and ∝*, c* are the kernel parameters.

For our study, we used a different kernel-based multiclass SVM from Scikit-learn 0.19.2 library (Pedregosa et al., 2011). Scikit-learn library internally use LIBSVM (Chang and Lin, 2011) to handle all computations. The hyperparameter of the kernel-based SVM must be tuned to measure how much maximum performance can be augmented by tuning it. It is important because they directly control the behavior of the training algorithm and have a significant impact on the performance of the model is being trained. Moreover, a good choice of hyperparameter can really make an algorithm smooth. Therefore, to find an optimal hyperparameter for the kernel-based multiclass SVM, *C* (explains the SVM optimization and percentage of absconding the misclassified trained data. For high *C* values, training data will classify accurately by a hyperplane; similarly, for low *C* values, optimizer looks for a higher margin separating hyperplane while it will misclassify the more data points) and γ (Gaussian kernel width describes the impact of specific training data. The high gamma values result in consideration of datasets that are near to separation line. Likewise, for low gamma values, datasets that are away from the separation line, will also be taken into consideration while in the calculation line) parameters are optimized using a grid search and a ten-fold stratified cross-validation (CV) method on the training dataset. This validation technique gives an assurance that our trained model got most of the patterns from the training dataset. Moreover, CV works by randomly dividing training dataset into 10 parts, one of which was left as a validation set, while the remaining nine were used by a training set. In this study, ten-fold stratified cross-validation was performed 100 times to obtain more accurate results. Finally, we computed the arithmetic mean of the 100 repetitions as the final result. Note that, as a different feature had different scales, so in our case, we linearly ascend each training feature to imitate to a range between 0 and 1; the same scaling technique was then applied to the test dataset. As the number of selected features is small, in our case the RBF kernel performs better than other kernels.

### Measuring the Classification Performance

To assess the classification performance of each group we have applied two method: (i) ROC-AUC curve analysis and (ii) Statistical analysis using Cohen's kappa method.

#### ROC-AUC Analysis

The ROC-AUC is a fundamental graph in the evaluation of diagnostic tests and is also often used in biomedical research to test classification problem performance and prediction models for decision support, prognosis, and diagnosis. ROC analysis examines the accuracy of a proposed model to separate positive and negative cases or distinguish AD patients from different groups. It is particularly useful in assessing predictive models since it records the trade-off between specificity and sensitivity over that range. In a ROC curve, the true positive rate (known as the sensitivity) is arranged as a function of a false positive rate (known as the 1-specificity) for different cut-off values of parameters. Each point's level of the ROC curve characterizes a sensitivity/specificity pair, which corresponds to a specific decision threshold. This is generally depicted in a square box for convenience and it's both axes are from 0 to 1. The area under curve (AUC) is an effective and joint measure of sensitivity and specificity for assessing inherent validity of a diagnostic test. AUC curve shows us how well a factor can differentiate between two binary diagnostic groups (diseased/normal). A result with perfect discrimination has a 100% sensitivity, 100% specificity ROC curve. Therefore the closer the ROC curve to the upper left corner, the higher the overall accuracy of the test as suggested by Greiner et al. (2000). The AUC is commonly used to visualize the performance of binary classes, producing a classifier with two possible output classes. Accuracy is measured using the AUC. Here, an AUC of one signifies a perfect score, and an area of 0.5 represents a meaningless test.

The AUC plot provides two parameters:

1. True positive rate (TPR): the TPR is a performance measure of the whole positive part of a dataset.
2. False positive rate (FPR): the FPR is a performance measure of the whole negative part of a dataset.

Moreover, classification accuracy measures the effectiveness of predicting the true class label, but in our case, it should be noted that the number of subjects was not the same in each group, so only calculating accuracy may result in a misleading estimation of the performance. Therefore, four more performance metrics have been calculated, namely specificity, sensitivity, precision, and F1-score. We have reported the accuracy, specificity, sensitivity, precision, and F1-score values corresponding to the ideal point of the ROC curve.

(11) $$\begin{array}{l} Accuracy=\frac{TP\;+\;TN}{TP\;+\;FP\;+\;FN\;+\;TN} \end{array}$$

(12) $$\begin{array}{l} F1-score=2*\left[\;\frac{precision*recall}{precision\;+\;recall}\;\right] \end{array}$$

where,

(13) $$\begin{array}{l} Precision=\;\frac{TP}{TP\;+\;FP}\;;\;Recall=Specificity=\;\frac{TP}{TP\;+\;FN} \end{array}$$

With TP, FP, TN, and FN denoting true positive, false positive, true negative, and false negative, respectively. Specificity (true negative rate) provides a amount for those not in the class, i.e., it is the percentage of those not in the class that were found not to be in the class. Precision [which is also termed as positive predictive value (PPV)] is the fraction of relevant incidences among the retrieved incidences, and F1-score (which is also called F-score or F-measure) is a amount related to a test's accuracy. Moreover, in our case, we have repeated the procedure 100 times, the reported AUC-ROC, accuracy, sensitivity, specificity, precision, and F1-score are the average over the 10 repetitions of the 10-fold stratified cross-validation procedure. We have followed this method for every classification groups.

#### Statistical Analysis Using Cohen's Kappa Method

Cohen's kappa statistic value for each classification problem was computed. This function calculates Cohen's kappa score, which demonstrate the level of agreement between two annotators or the level of agreement between two dissimilar groups in a binary classification problem defined as,

(14) $$\begin{array}{l} k=\left(p_{o}-p_{e}\right)/\left(1-p_{e}\right) \end{array}$$

where, *p*_*o*_ is the empirical probability of an agreement on the label assigned to any example (the observed agreement ratio), and, *p*_*e*_ is the predictable agreement when both annotators assign labels randomly. Here, *p*_*e*_ was assessed using a per-annotator empirical prior over the class labels. The kappa statistic value is always between −1 and 1. The maximum value means complete agreement between two groups, zero or lower value means a low probability of agreement.

## Results

Here, all classification problems were performed using Ubuntu 16.04 LTS, running python 3.6, and using Scikit-learn library version 0.19.2. In this study, there were four classes of data, AD, MCIc, MCIs, and HC, separated using four different types of biomarker, sMRI and FDG-PET for imaging modalities, and CSF as a biochemical (or fluid vessel) that show results reflecting the formation of amyloid plaques inside the brain, and APOE genotypes as genetic features. Thus, we validated our proposed method on six different types of classification problem, i.e., six binary class problem (AD vs. HC, MCIc vs. MCIs, AD vs. MCIc, HC vs. MCIs, HC vs. MCIc, and AD vs. MCIs). At first, we extracted the featured from each sMRI and FDG-PET images by using the NiftyReg registration process with 2-mm Brainnetome atlas template image. In total, we obtained 497 features for a single image, 246 ROI-based features from the sMRI and FDG-PET images, three feature values from the CSF data, and two features from the APOE genotype data. Moreover, we have applied a random tree embedding method which transformed obtained low dimension features into a higher dimensional state, after that an early fusion technique is processed to combine the multiple features into single form before passing them for further process. Additionally, we have also applied a feature selection technique using a truncated SVD dimensionality reduction method, which will select the effective features from all 497 high dimensional features and send these selected features to the classifier, to measure the performance of identifying each group. In our case, we used a kernel-based multiclass SVM as a classifier from a Scikit-learn library (0.19.2).

In order to attain unbiased estimates of performance, the set of participants were randomly split into two groups in a 75:25 ratios as training and testing sets, respectively.

**Figure 4:**
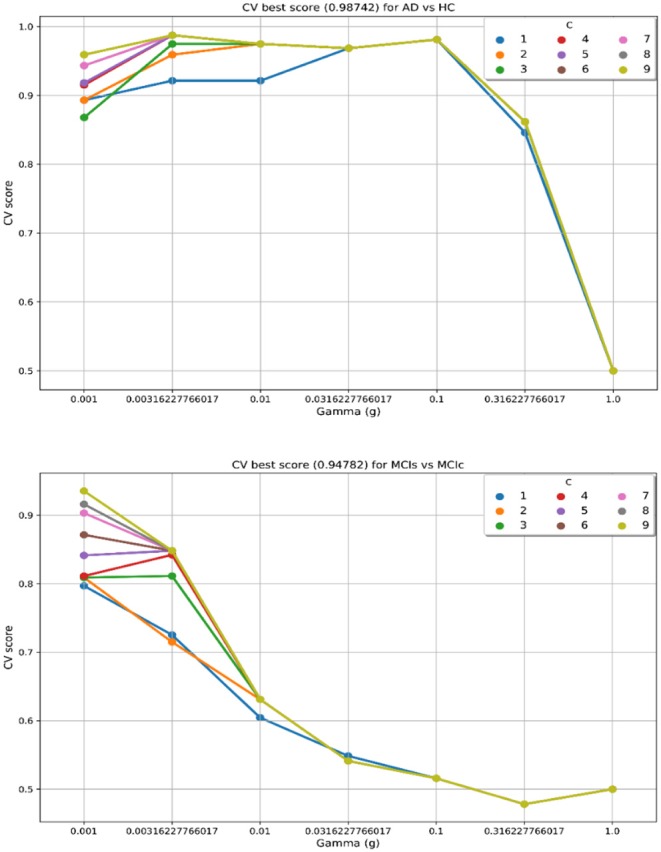
CV best score obtained for AD vs. HC and MCIs vs. MCIc groups. Best CV score is computed by taking the average of 10 folds CV values. CV score, Cross-validation score; *C*, regularization constant; and *g or* γ, gamma, *C* and γ are the hyperparameter value for the kernel-based multiclass SVM.

In the training set, to find the right values for the hyperparameter (*C* and γ) is very difficult, and their values influence the classification result. Moreover, we know that the parameter *C*, trades off the misclassification of training samples against the simplicity of a decision surface, a small *C* value makes the decision surface flat, while a high *C* value aims to classify all training samples correctly. Moreover, a γ value shows how much influence a single training sample has. The larger γ is, the closer other samples must be to be affected. Therefore, we have used cross-validation technique to get good optimal hyperparameter values for the regularization constant (*C*) and gamma (γ). We can't know the best value for a model hyperparameter on a given problem. With the right values of hyperparameters will eliminate the chances of overfitting and underfitting. Therefore, to find the optimal hyperparameter values for a kernel-based SVM, have used a grid-search (which perform a comprehensive search over the specified parameter values for an estimator) and ten-fold stratified cross-validation technique on the training set. The grid search was performed over the ranges of *C* = 1 to 9 and γ = 1e-4 to 1. For each method, the gained optimized value of the hyperparameter was then used to train the classifier using the training set, and later the performance of the resulting classifier was then evaluated on the remaining 25% of data in the testing dataset, which was not used during the training step. The obtained optimized hyperparameter (*C* and γ) value and their best CV accuracy are shown in Table 3. Figure 4 is a plot of the classifier's CV accuracy with respect to (*C* and γ) for AD vs. HC and MCIc vs. MCIs groups. In Figure 4, we can see the impact of having different *C* and γ values on the model. Moreover, the best found optimal hyperparameter combination for an AD vs. HC are *C* = 7, γ = 0.00316227766017 and for MCIs vs. MCIc are *C* = 9, γ = 0.001, these tuned optimal hypermeter values are automatically chosen from the given range of *C* = 1 to 9 and γ = 1e-4 to 1 with the help of grid search and ten-fold CV. In this way, we achieved unbiased estimates of the performance for each classification problem. In our experiment, the number of subjects was not the same in each group. Therefore, only calculating accuracy does not enable a comparison of the performances of the different classification experiments. Thus, we have considered five measures. For each group, we have calculated the accuracy, sensitivity, specificity, precision, and F1-score performance measure values. Table 4 show the classification results for AD vs. HC, MCIc vs. MCIs, AD vs. MCIs, AD vs. MCIc, HC vs. MCIc, and HC vs. MCIs.

**Table 3:** Obtained best CV score for six classification groups.

**Group**	**Regularization constant (c)**	**Gamma (g or γ)**	**Best CV score**
AD vs. HC	7	0.00316227766017	0.98742
AD vs. MCIs	5	0.01	0.96207
AD vs. MCIc	6	0.004	0.92201
MCIs vs. MCIc	9	0.001	0.94782
HC vs. MCIc	4	0.001	0.94036
HC vs. MCIs	9	0.001	0.93103

**Table 4:** Classification results for AD vs. HC, MCIs vs. MCIc, AD vs. MCIs, AD vs. MCIc, HC vs. MCIc, and HC vs. MCIs groups.

**Groups**	**Features**	**Classifier**	**Performance measure**						
			**AUC**	**ACC**	**SEN**	**SPEC**	**PRE**	**F1-score**	**Cohen's kappa**
AD vs. HC	APOE genotype	SVM	90.83	88.96	90.91	89.46	86.92	87.33	0.79
	CSF		91.54	91.3	88.48	91.67	90.91	93.15	0.82
	sMRI		93.33	92.5	100	89.74	88.47	92.86	0.85
	FDG-PET		92.5	92.56	89.74	90	93.62	91.63	0.84
	Combined		**98.33**	**98.42**	**100**	**96.47**	**97.89**	**98.42**	**0.93**
MCIs vs. MCIc	APOE genotype	SVM	91.21	92	86.67	**100**	**100**	**92.86**	0.83
	CSF		87.73	88	85.71	90.91	92.31	88.89	0.75
	sMRI		86.54	85.43	84.55	83.92	86.96	81.82	0.69
	FDG-PET		90.38	89	100	85	76.92	86.96	0.76
	Combined		**93.59**	**94.86**	**100**	88.71	89.62	91.67	**0.86**
AD vs. MCIs	APOE genotype	SVM	90	89.96	100	82.73	84	88.89	0.75
	CSF		94.17	93.33	91.3	**100**	86.67	92.86	0.86
	sMRI		88.33	87.67	82.61	86.49	73.33	84.62	0.73
	FDG-PET		89.17	90	89.96	91.73	89.96	88.89	0.75
	Combined		**96.83**	**96.65**	**100**	91.67	**93.33**	**96.55**	**0.91**
AD vs. MCIc	APOE genotype	SVM	88.89	88.46	77.78	**94.12**	87.2	82.35	0.71
	CSF		89.58	86.39	86.92	90	87.5	88.67	0.73
	sMRI		84.52	80.36	80.77	81.82	80	78.26	0.69
	FDG-PET		84.03	80.77	66.67	88.24	75	70.59	0.65
	Combined		**94.64**	**92.26**	**91.67**	92.86	**91.67**	**91.67**	**0.84**
HC vs. MCIc	APOE genotype	SVM	87.5	87.12	82.64	87.5	86.67	92.33	0.73
	CSF		94.05	92.31	91.67	90.44	92.26	95.22	0.83
	sMRI		89.58	88.46	90.91	86.67	83.33	86.96	0.76
	FDG-PET		91.07	87.5	88.46	100	82.35	85.71	0.76
	Combined		**96.43**	**94.13**	**94.75**	**100**	**100**	**96.72**	**0.88**
HC vs. MCIs	APOE genotype	SVM	90.83	87.08	86.96	**92.86**	86.67	89.66	0.72
	CSF		92.5	90.47	100	72.73	80	88.89	0.73
	sMRI		91.27	90.16	98.26	86.67	90.91	92	0.71
	FDG-PET		89.68	87.3	92.31	80	85.71	88.89	0.73
	Combined		**95.24**	**95.65**	**100**	88.89	**93.33**	**96.55**	**0.90**

We conduct the AD vs. HC experiment using extracted APOE, CSF, FDG-PET, and sMRI features, and the classification outcome is shown in Table 4. For AD vs. HC classification, we had 38 AD and 38 HC subjects and only sMRI individual biomarker performed well while compared to other individual modalities of biomarkers. Moreover, the early fusion technique that we used to combine features from different modalities resulted in an AUC of 98.33, 98.42% of accuracy, 100% of sensitivity, 96.47% of specificity, 97.89% of precision, and 98.42% of F1-score. Furthermore, Cohen's kappa value is 0.93 for the combined method, which is very close to 1. Likewise, for the MCIs vs. MCIc classification problem, 82 subjects were included. Forty-six were in the MCIc group and the remaining 36 patients were in the MCIs group. Table 4 shows the computed performance measure for this classification problem. Compared to other classification group problem this classification group (MCIs vs. MCIc) is difficult to classify because both groups show similar brain structure; however, there are slight differences in structure. For this group, APOE genotype individual biomarker performed well while compared to other individual modalities of biomarkers. Moreover, our proposed method has performed even better than the best output obtained by individual biomarkers for this group and the achieved measures are AUC of 93.59%, with 94.86% accuracy, 100% sensitivity, 88.71% specificity, 89.62% precision, and an F1-score of 91.67% compared to those of the single modalities. For MCIs vs. MCIc, Cohen's kappa value was 0.86, which is better than those of the single modalities. Our proposed method has performed very well when classifying this group. For AD vs. MCIs group, there were 38 AD and 36 MCIs subjects. First, we extracted the features from each subject and then we combined both imaging (PET and MRI) feature values with the other two (CSF and APOE genotype) feature values to measure the performance of AD vs. MCIs classification. Table 4 shows the results from passing obtained features to the kernel-based multiclass SVM classifier. As can be seen from Table 4, our proposed method to combine all four modalities of a biomarker for distinguishing between AD and MCIs achieved good results compared to single modality biomarkers. For this classification problem, our proposed method achieved 96.65% of accuracy with a Cohen's kappa of 0.91. For AD vs. MCIc group, there were 38 AD and 46 MCIc. We trained kernel-based multiclass SVM classifiers using dimensionality-reduced features from truncated SVD to measures the performance of AD vs. MCIc group. The best performance was attained using a combination of four modalities of features, i.e., sMRI, FDG-PET, APOE and CSF, which had an accuracy of 92.26%, a sensitivity of 91.67%, a specificity of 92.86%, and an AUC of 94.64% with Cohen's kappa of 0.84. For the HC vs. MCIc distinction, our proposed method achieved 96.43% AUC, 94.13% accuracy, 94.75% sensitivity, 100% of specificity and precision, and 96.72% of F1-score. Table 4 shows the classification performance result for HC vs. MCIc classification. In this case, the obtained Cohen's kappa index value is 0.88, which is near to the maximum level agreement value of 1. For the HC vs. MCIs classification problem, 74 subjects were included. Thirty-six were in the MCIs group and the remaining 38 patients were in the HC group. Table 4 shows the results from passing obtained features to the kernel-based multiclass SVM classifier. As can be seen from Table 4, our proposed method to combine all four modalities of a biomarker for distinguishing between HC and MCIs achieved good results compared to single modality biomarkers. For this classification problem, our proposed method had achieved 95.24% of AUC, and 95.65% of accuracy with a Cohen's kappa of 0.90. Therefore, we can say that for all classification groups our proposed method has achieved a high level of performance while compared to single modality of biomarkers, ranging from 1 to 5%, and our proposed method has also achieved a high level of agreement between each other for all six classification groups while compared with single modality-based methods. For AD vs. MCIs, AD vs. MCIc, HC vs. MCIs, and HC vs. MCIc groups, CSF individual biomarkers performed very well-compared to other individual modality of biomarkers, and the CSF achieved AUC for these groups are 94.17, 89.58, 94.05, and 92.5%.

**Figure 5:**
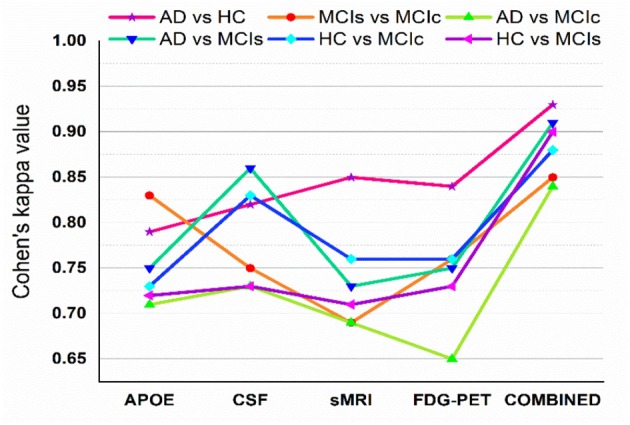
Cohen's kappa score for AD vs. HC, MCIs vs. MCIc, AD vs. MCIc, AD vs. MCIs, HC vs. MCIs, and HC vs. MCIc (each experiment obtained kappa value is shown by different solid color lines). This Cohen's kappa plot shows that combined features outperform the single modality features in all experiments.

Figure 5 shows Cohen's kappa statistics score for six classification problems, AD vs. HC, MCIs vs. MCIc, AD vs. MCIs, AD vs. MCIc, HC vs. MCIs, and HC vs. MCIc. From this graph, we can see that our proposed method has achieved a good level of agreement between different classification groups.

**Figure 6:**
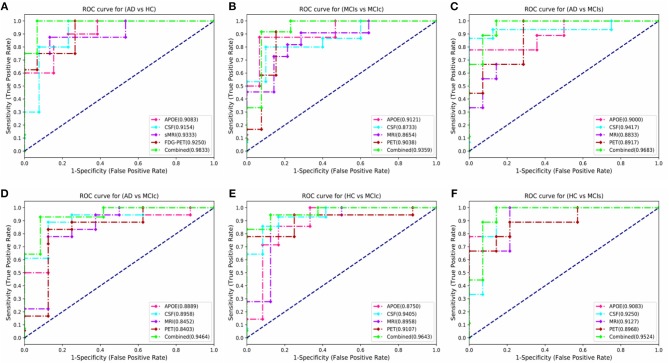
Comparison of the ROC-AUC curve corresponding with the best performance of combined fusion method in each experiment is displayed by the green dashed lines. We also compare these ROC-AUC curves with those of single modality features. This comparison shows that combined features outperform the single modality features in all experiments, which can be seen from above figure **(A)** AD vs. HC, **(B)** MCIs vs. MCIc, **(C)** AD vs. MCIs, **(D)** AD vs. MCIc, **(E)** HC vs. MCIc, and **(F)** HC vs. MCIs.

Here, Figure 6 shows the AUC curve for AD vs. HC, MCIs vs. MCIc, AD vs. MCIs, AD vs. MCIc, HC vs. MCIs, and HC vs. MCIc. Total AUC-ROC curve is a single index for measuring the performance of a test. The larger the AUC, the better is the overall performance of the diagnostic test to correctly pick up diseased and non-diseased subjects. For AD vs. HC, our proposed model achieved 98.33% AUC, showing that our proposed model performed well when distinguishing positive and negative values. For MCIs vs. MCIc, our proposed model correctly distinguished converted patients from stable patients with an AUC of 93.59%, which is a great achievement for this complex group. Likewise, for AD vs. MCIs, AD vs. MCIc, HC vs. MCIs, and HC vs. MCIc, our proposed model achieved AUCs of 96.83, 94.64, 95.24, and 96.43%. Overall, for all classification methods, our proposed model performed well and its probabilities for the positive classes are well-separated from those of the negative classes.

## Discussion

In this experiment, we proposed a novel technique to fuse data from multiple modalities for the classification of AD from different groups, using a kernel-based multiclass SVM method. In addition, earlier studies aimed only for AD vs. HC classification groups. In this paper, we studied six binary classification problem, AD vs. HC, MCIs vs. MCIc, AD vs. MCIs, AD vs. MCIc, HC vs. MCIs, and HC vs. MCIc. More importantly, we combined not only sMRI and FDG-PET images but also their CSF (biochemical) and APOE (genetic) genotype values. Our experiment result shows that each modality (sMRI, FDG-PET, CSF, and APOE) is indispensable in achieving good combination and good classification accuracy.

Some studies (Zhang et al., 2011, 2012; Young et al., 2013) have used a small number of features extracted from automatic or manual segmentation processes for the classification of AD from different groups. Their proposed model has achieved good performance for AD vs. HC; however, for MCIc vs. MCIs, the performance of their proposed model is poor. Therefore, in our study, we tried to extract as many ROI from two imaging modalities using the 2-mm Brainnetome template image. To the best of our knowledge, this is the first experiment where 246 ROI was extracted from all 158 subjects and all features were used in the classification of AD and MCI subjects.

Furthermore, we later fused features from these two imaging modalities with three CSF and two APOE genotype features offered by the ADNI website for the distinction of AD from different groups using early fusion technique. Moreover, we use a more advanced segmented template image for feature extraction from both imaging modalities with the NiftyReg registration toolbox, compared to other studies (Walhovd et al., 2010; Davatzikos et al., 2011; Zhang et al., 2011; Beheshti et al., 2017; Li et al., 2017; Long et al., 2017). As we can see that from Table 4, single modality biomarkers (sMRI and APOE genotype) achieved a good performance for AD vs. HC and MCIs vs. MCIc (using all 246 extracted features and as well as with two APOE genotype feature from each subject) groups, when compared with the obtained outputs reported before (Zhang et al., 2011; Young et al., 2013). Likewise, from same Table 4, we can see that CSF individual modality of biomarkers has outperformed other individual biomarkers with 94.17, 89.58, 94.05, 92.5% of AUC for AD vs. MCIs, AD vs. MCIc, HC vs. MCIc, and HC vs. MCIs. Moreover, a lot of studies have shown that different modalities of biomarkers contain complementary information for the discrimination of AD and MCI subjects. Here, we quantitatively measure the discrimination agreement between any two different classification groups using the kappa index. For combined features (for AD vs. HC, AD vs. MCIs, and AD vs. MCIc), the obtained level of agreement between each group is 0.93, 0.91, and 0.84, respectively. Likewise, for HC vs. MCIs and HC vs. MCIc, the obtained level of agreement between each group are 0.90 and 0.88. Moreover, for MCIs vs. MCIc group the obtained level of agreement between each other is the 0.86, respectively. These all scores are achieved using a 10-fold stratified CV method on combined dataset. These results indicate that the combined feature (for AD vs. HC) group has the highest level of agreement between each other while compared to other groups and as well as while compared to the individual performance of each modality.

**Figure 7:**
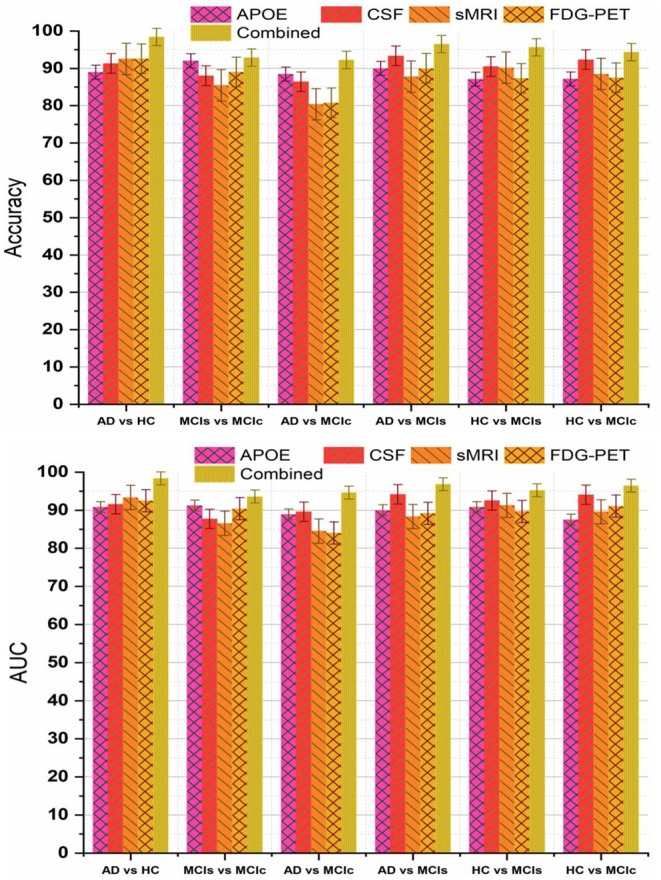
Comparison of single modality results with multi-modality classification result based on obtained accuracy and AUC score. From above figure we can see that in every group combined feature (or multimodal method) has outperformed the single modality results.

Recently, many studies have been published using a single modality of biomarkers (Chetelat et al., 2007; Fjell et al., 2010; Chen and Ishwaran, 2012; Beheshti et al., 2016; Jha et al., 2017; Lama et al., 2017; Long et al., 2017), including sMRI, FDG-PET, CSF, and APOE. Most of these studies used biomarkers from the sMRI, because it is practically difficult to get biomarkers from all modalities for the same patients due to the various reasons, including the availability of imaging equipment, cost, lack of patient consent, and patient death in longitudinal studies. Previously proposed models using a single modality have achieved good performance for AD vs. HC classification, where for MCIs vs. MCIc their classification accuracy is very low compared to our proposed multimodal technique. Here, we have performed an experiment to assess the classification performance using features from every single modality independently, as well as with the combination of multimodal biomarkers. A kernel-based multiclass SVM classifier was utilized, and the comparison of the obtained single modality results with the multimodal classification results are shown in Figure 7. In terms of accuracy and AUC, the classification performance using features from CSF is generally better than those using genetic and imaging features, which highlights the importance of Aβ plaques as biomarkers in the classification of AD, while in comparison to the performance with multimodal biomarkers, its performance is slightly lower. In addition, we can see that for the MCIs vs. MCIc comparison, each modality of biomarker has performed well. Different methods were used to evaluate the classification of AD using multimodal data. First, we combine all high-dimensional features from four modalities into a single feature vector for classification of AD and MCI subjects. After that, all features were normalized (to have a zero mean ± unit standard deviation) before using them in the classification process. This combined multimodal method provides a straightforward method of using multimodal data. Subsequently, we passed these features to the kernel-based multiclass SVM classifier for classification purposes with a 10-fold stratified CV strategy as described above, and obtained results are shown in Table 4 and Figure 7. As we can see in Table 4, our early fusion combination method consistently outperforms the performance of individual modality of biomarkers.

Recently, several studies have investigated neuroimaging techniques for the early detection of AD, with the main focus on MCI subjects, who may or may not convert to AD, and separating patients with AD from healthy controls using multimodal data. However, it is difficult to make direct comparisons with these state-of-the-art methods since a majority of the studies have used different validation methods and datasets, which both crucially influence the classification problem. The first study by Zhang et al. (2012) obtained an accuracy of 76.8% (sensitivity and specificity of 79 and 68%) for the classification of converters and stable MCI subjects within 24 months.

These results were achieved using a multi-kernel SVM on a longitudinal ADNI dataset. Another study (Young et al., 2013) used a Gaussian process method for classification of MCIs vs. MCIc using several modalities. They reported an accuracy of 69.9% and AUC of 79.5%. Another study by Suk et al. (2014) used shared features from two imaging modalities, MRI and PET, using a combination of hierarchical and deep Boltzmann machines for a deep learning process; their proposed method achieved 74.66% accuracy and 95.23% AUC when comparing MCI-C vs. MCI-NC. In another study (Cheng et al., 2015), the authors introduced domain transfer learning using multimodal data (i.e., MRI, CSF, and PET) with an accuracy of 79.4% for MCIs vs. MCIc with an AUC of 84.8%. In another study (Moradi et al., 2015), the authors employed a VBM analysis of gray matter as a feature, combining age and cognitive measures. They reported an AUC of 90.20% with 81.72% accuracy comparing MCIc vs. MCIs sample. Another study (Beheshti et al., 2017), used feature ranking and a genetic algorithm (GA) for selection of optimal features for the classifier. Their method achieved an accuracy of 75%, sensitivity of 76.92%, specificity of 73.23%, and AUC of 75.08% for pMCI vs. sMCI. Liu et al. (2017) proposed combining two imaging modalities using independent component analysis and the Cox model for prediction of MCI progression. They achieved 80.8% AUC with 73.5% accuracy in comparisons of MCIc vs. MCIs. Recently, another author (Long et al., 2017) used Free surfer software to segment 3-T T1 images into many different parts and later used a multi-dimensional scaling method for feature selection before sending the selected features to the classifier. Their proposed method achieved an AUC of 93.2%, accuracy of 88.88%, sensitivity of 86.32, and specificity of 90.91% when differentiating sMCI from pMCI using only specific amygdala features. As shown in Table 5, the performance of the proposed system was highly competitive in performance terms when compared to the other systems reported in the literature for MCIs vs. MCIc classification.

**Table 5:** Classification performance for the proposed method compared with published state-of-the art methods for differentiating between MCIs vs. MCIc.

**Method**	**Modality**	**Subjects**	**AUC**	**ACC**	**SEN**	**SPEC**
Zhang et al. (2012)	Longitudinal (MRI + PET)	88	76.8	78.4	79	68
Young et al. (2013)	MRI + PET + APOE	143	79.5	69.9	78.7	65.6
Suk et al. (2014)	PET + MRI	204	74.66	75.92	48.04	**95.23**
Cheng et al. (2015)	MRI + PET + CSF	99	84.8	79.4	84.5	72.7
Moradi et al. (2015)	MRI + AGE + Cognitive measure	264	90.20	81.72	86.65	73.64
Beheshti et al. (2017)	MRI	136	75.08	75	76.92	73.23
Liu et al. (2017)	MRI + PET	234	80.8	73.5	76.19	70.37
Long et al. (2017)	MRI (AMYG)	227	93.2	88.99	86.32	90.91
Proposed method	MRI + PET + CSF + APOE genotype	82	**93.59**	**94.86**	**100**	88.71

## Conclusion

In this study, we have proposed a novel method that shows how to extract 246 ROI from two imaging modalities, PET and sMRI, using a Brainnetome template image and then combined these features obtained from imaging with CSF and APOE genotype features from the same subjects. In the proposed method, we used a random tree embedding method to transform obtained features to a higher dimensional state and later we used a truncated SVD dimensionality reduction method to select only the important features, which increased the classification accuracy using kernel-based multiclass SVM classifier. The obtained experimental results prove that a combination of biomarkers from all four modalities is a reliable technique for the early prediction of AD or prediction of MCI conversion, especially with regards to high-dimensional data pattern recognition. In addition, our proposed method achieved 94.86% accuracy with 93.59% AUC and a Cohen's kappa index of 0.86 when distinguishing between MCIs vs. MCIc subjects. The performance of the proposed computer-aided system was measured using 158 subjects from the ADNI dataset with a 10-fold stratified cross-validation technique. The experimental results show that the performance of the proposed approach can compete strongly with other state-of-the-art techniques using biomarkers from all four modalities mentioned in the literature.

In future, we plan to combine demographic information of the studied subjects as features with the proposed model for the classification of AD and we will also carry out an investigation of the multimodal multiclass classification of AD using AV-45 and DTI modality of biomarkers.

## Data Availability Statement

The dataset used in this study were acquired from ADNI homepage, which is available freely for all researcher and scientist for experiments on Alzheimer's disease and can be easily downloaded from ADNI websites: http://adni.loni.usc.edu/about/contact-us/.

## Author Contributions

YG and RL designed the study, collected the original imagining data from ADNI home page, and wrote the manuscript. RL and G-RK managed and analyzed the imaging data. All authors contributed to and have approved the final manuscript.

### Conflict of Interest

The authors declare that data used in preparation of this article were obtained from the Alzheimer's Disease Neuroimaging Initiative (ADNI) database (adni.loni.usc.edu). As such, the funder and the investigators within ADNI contributed to the data collection, but did not participate in analysis, interpretation of data, the writing of this article or the decision to submit it for publication.
